# Sodium Channel Blockers for Vestibular Paroxysmia in Children

**DOI:** 10.3390/audiolres15030065

**Published:** 2025-06-05

**Authors:** Pierre Reynard, Hung Thai-Van, Eugenia Mustea, Alexandra Neagu, Samar A. Idriss, Eugen C. Ionescu

**Affiliations:** 1 Department of Audiology and Otoneurological Explorations, Civil Hospitals of Lyon, 69003 Lyon, France; hung.thai-van@chu-lyon.fr (H.T.-V.); eugenia.mustea@chu-lyon.fr (E.M.); eugen.ionescu@chu-lyon.fr (E.C.I.); 2 Department of Physiology, Claude Bernard Lyon 1 University, 69003 Lyon, France; 3 Paris Hearing Institute, Institut Pasteur, Inserm U1120, 75015 Paris, France; 4 Department of Otorhinolaryngology, MS Curie Emergency Children Hospital, 077120 Bucharest, Romania; aaaneagu30@yahoo.com; 5 Department of Otolaryngology, Dar Al Shifa Hospital, Hawally 13034, Kuwait; 6 Department of Otolaryngology and Head and Neck Surgery, Holy Spirit University of Kaslik, Eye and Ear Hospital, Beirut 1201, Lebanon; 7 Department of Otorhinolaryngology, Emergency University Hospital, 050098 Bucharest, Romania

**Keywords:** vestibular paroxysmia, sodium channel blockers, cross compression syndrome, narrowed internal auditory canal, carbamazepine, oxcarbazepine

## Abstract

**Background/Objectives:** As vestibular paroxysmia (VP) has recently been described in children, with an incidence of up to 4% of vertigo, and a promising therapeutic response to sodium-channel-blocking drugs has also been reported, the aim of this paper is to review the available literature on this topic and to provide the best possible guidance for diagnosis and treatment. **Methods**: PubMed, Medline, Cochrane, and Crossref databases were searched, and all studies on VP in children and sodium channel blockers were selected. **Results**: Only five articles reporting small case series or single case reports were identified. To date, oxcarbazepine (OXC) and carbamazepine (CBZ) are the only two molecules prescribed. The recommended doses were 300 to 360 mg/day and 50 to 200 mg/day for OXC and CBZ, respectively, for a total duration of 6 weeks. Fast efficacy (one week) was reported. **Conclusions**: VP has been identified in pediatric patients and appears to respond to sodium channel blockers in a manner similar to adults. Only a limited number of cases have been reported to date; thus, there is a need to raise awareness about this treatable cause of episodic vertigo in children.

## 1. Introduction

Paroxysmal sensory or motor attacks in the territories innervated by cranial nerves are usually caused by direct pressure of blood vessels on the cisternal portion of the respective nerves. These clinical entities are known as Neurovascular Cross Compression Syndromes (NVCCSs). They mainly include trigeminal neuralgia, hemifacial spasm, glossopharyngeal neuralgia, and vestibular paroxysmia (VP) [1]. Neurovascular compression can be visualized using MRI and may lead to a demyelination process and/or ephaptic discharges. However, evidence suggests that these phenomena may also occur in the absence of any underlying anatomopathological conditions, such as in NVCCSs [2,3]. When the cochleovestibular nerve (CVN) is involved, the patient presents with recurrent, brief, stereotyped, and likely positional vertigo. The term VP, formerly known as disabling positional vertigo, was initially identified during surgical interventions in patients presenting the latter-mentioned symptoms, concomitant with a direct neurovascular compression of the CVN [4,5]. Subsequently, clinical diagnostic criteria for definite VP were published by the Bárány Society: at least 10 attacks of stereotyped, short spontaneous vertigo (<1 min) in the absence of a better explanation by another diagnosis; interestingly, response to treatment with sodium channel blockers, such as carbamazepine (CBZ) or oxcarbazepine (OXC), was included among the diagnostic criteria [6].

A clinical context of VP associated with radiologic (MRI) findings suggestive of NVCCSs is usually considered a formal cause of VP. According to Rommer et al., symptoms of VP are not associated with structural nerve lesions and could be induced by simple contact between a vascular structure and the CVN (or possible mild structural abnormalities that are yet undetectable on 7T MRI) [7]. The pathophysiology of VP has already been discussed elsewhere [8]. Briefly, pressure on the nerve could provoke localized neuropathy with a local ephap-like mechanism; inhibitory and activating potentials could be generated, affecting the normal frequency of action potentials, leading to desynchronised nerve activity. The pathogenesis of VP may also result from a combination of transient vestibular inhibition secondary to conduction blocks and paroxysmal vestibular excitations induced by head movements.

Studies have shown the absence of a systematic association of radiologic NVCCSs among VP patients [2,3]. Additionally, the presence of a narrowed internal auditory canal (IAC; container) with a normally sized cochleovestibular nerve (CVN; content) has been described in both children and adults with symptoms suggestive of VP, without imaging evidence of NVCCSs [9,10,11]. This entity could, in some patients, explain the presence of VP symptoms in individuals without evidence of NVCCSs. The neuropathy appeared to be less focal than in NVCCSs and was reminiscent of other canalar syndromes [9,10,11]. Recently, VP has been classified, similar to trigeminal neuralgia, into classic VP (type I) due to NVCCSs, secondary VP (type II) due to a “non-vascular” compression (osteoma, schwannoma, narrowed IAC), and idiopathic VP (type III) [8]. Sodium channel blockers were used in VP cases without clear evidence of NVCCSs, with positive outcomes, thus questioning the link between VP and the presence of NVCCSs and raising the question of the presence of another cause [3]. Consumption of these drugs proved to be efficient in both type I and type II VP by reducing clinical symptoms and normalizing electrophysiological findings [9,10,11], which is one of the main diagnostic criteria for definite VP [6]. In adults, treatment with sodium channel blockers was recommended based on a small case series with low level of evidence [6]. They included CBZ, OXC, and other less-prescribed molecules. Voltage-gated sodium channels regulate membrane excitability, which has a significant role in various physiological and neuronal processes.

VP has recently been described in children, with comparable clinical features and favorable therapeutic responses [11,12,13]. According to Jahn et al., VP accounts for up to 4% of vertigo in children [14]. The present review aims to summarize the available evidence on the treatment of VP in children using sodium channel blockers. It also provides a reliable assessment of the current state of knowledge about VP diagnosis and therapy.

## 2. Materials and Methods

### 2.1. Search Strategy

A computerized literature search was conducted on PubMed^®^, Medline, Cochrane, and Crossref platforms to identify recent, relevant publications in peer-reviewed journals. The search terms “vestibular paroxysmia” and “treatment” were combined. Studies published until March 2025 were included. The main point of focus was on studies using sodium channel blockers in the management of VP in the pediatric population.

### 2.2. Study Selection

All published studies on VP in the pediatric population treated with sodium channel blockers were selected. Studies in which treatment was evaluated using precisely described intervention protocols, over a short or long duration, were eligible for inclusion. Duplicates were excluded. In view of the paucity of literature on the topic, all types of articles (including case reports and small case series) were included. The review was not limited to English-language publications and included articles written in other languages.

## 3. Results

### 3.1. Search Strategy and Study Selection

PubMed and Medline searches for articles published until October 2024 using the previously mentioned keywords yielded 89 results. A total of 27 articles were excluded because they did not fall within the scope of the study: two animal studies, 22 addressed migraine or vestibular pathologies, and three involved surgical interventions; therefore, 62 articles were assessed. The eligibility assessment narrowed the selection tofour articles focusing on pediatric populations. Subsequent searches in the Cochrane and Crossref databases yielded only one additional article (in Korean), bringing the total number of included articles to five (Table 1). Notably, none of these articles presented a high level of evidence.

### 3.2. Data Extraction: Antiepileptic Drugs for VP

The included studies that assessed sodium channel blockers for all types of VP in children are presented in Table 1.

The first reported case of VP in a child, as described by Hong et al., involved a 7-year-old girl [13]. Her clinical history included isolated rotatory vertigo episodes, once or twice an hour, lasting approximately 10 s and occurring once or twice hourly over a 2-month period. These episodes were more prevalent during physical activity, such as running, and a right-sided nystagmus was noted concurrently. Pure tone audiometry (PTA) showed normal hearing thresholds. The caloric test demonstrated a 25% reduction in function of the left lateral semicircular canal. The hyperventilation provocation elicited a transient right-sided nystagmus lasting 5 s, which was subsequently followed by a left-beating nystagmus lasting 7 s. MRI identified compression of the CVN by the AICA within the left IAC. A one-week trial of Oxcarbazepine at 300 mg/day provided limited reduction (less than 50%) in vertigo episodes. Consequently, the dosage was increased to 360 mg/day, resulting in the cessation of symptoms within one week. The patient experienced a 5-month period without vertigo. However, the total length of Oxcarbazepine treatment was not specified.

Lehnen et al. investigated 400 children presenting with vertigo over a 10-year period, identifying 16 potential cases of VP with a mean age of 13 years [12]. However, their report detailed only three cases, all characterized by brief (seconds), repetitive episodes of vertigo throughout the day. Physical exertion triggered vertigo in two patients. All three reported oscillopsia, and one described instability. While PTA and vestibular assessment (including caloric, rotatory chair, and cervical vestibular evoked myogenic potentials) yielded normal results, hyperventilation-induced nystagmus (HVIN) was present in one patient. MRI consistently demonstrated NVCC of the CVN by the AICA in all three cases. Treatment with CBZ (50 to 100 mg/day or 3–4 mg/kg) proved effective in all 3 cases within a variable timeframe of 1 or more weeks. The duration of treatment ranged from several months upwards, and discontinuation attempts revealed varied success rates (1, 2, and 3 attempts for the three patients), with re-treatment consistently proving effective.

In a small cohort, 16 children with VP-like symptoms who were initially diagnosed with benign paroxysmal vertigo of childhood (BPVC) were included [11]. MRI excluded NVCCSs in half the cases. Instead, a potential narrowing of the IAC alongside normal cranial nerve morphology was noted. It was hypothesized that the cochleovestibular symptoms in these cases might arise from a localized entrapment neuropathy similar to carpal tunnel syndrome or radiculopathies caused by local compression. Recognizing that “Narrowed IAC” is a tomodensitometry term introduced by Valvassori, corresponding to a diameter of less than 2 mm (axial plane) with a hypoplastic or absent nerve [15], and given the preserved nerve morphology on MRI, the term “near” narrowed IAC (nNIAC) was proposed [9,10]. Eight children with nNIAC and eight children with VP by NVCCS were carefully selected to exclude confounding variables (ototoxicity, middle ear disease, neurological/ophthalmic disorders, psychological status, etc.). HVIN was positive (triggering of nystagmus, increase in spontaneous nystagmus) in 6 out of 16 children. Sodium channel blockers were prescribed for severe recurrent vertigo (assessed via DHI); four children in each group were treated with OXC. With the exception of one patient in the NVCCS group, all patients reported substantial subjective improvement upon starting medication. Short-term discontinuation (<3 months) led to vertigo recurrence in half of all patients. Notably, OXC effectively treated vertigo and normalized auditory brainstem I–III interpeak latencies in both nNIAC and NVCCS groups.

Nunez et al. described a 16-year-old child exhibiting symptoms consistent with VP according to Bárány Society criteria [16]. Despite MRI showing no NVCCSs on the CVN (though MRI details were lacking), and successful treatment of co-occurring Wolff-Parkinson-White (WPW) syndrome, the vestibular symptoms persisted. A prior COVID-19 infection and the failure of topiramate treatment (for suspected vestibular migraine) were also noted. Instead, the patient responded well to CBZ at a dosage of 200 mg/day (twice daily).

In another case report, a boy presented with paroxysmal non-rotary vertigo (around 10 daily episodes of a few seconds each) consistent with definite VP, at around the age of 18 [17]. The vertigo was not triggered by head movements or exertion, and audiovestibular findings (HVIN and PTA) were normal. Contrast-enhanced MRI of the brain identified a clear structural correlate: a congenital malformation of the AICA compressing the cisternal segment of the CVN. Treatment with carbamazepine (100 mg bid) for 2 months proved to be fully effective in abolishing the symptoms.

**Table 1:** Articles included in the review. VP: vestibular paroxysmia; CBZ: carbamazepine; OXC: oxcarbazepine; PTA: pure tone audiometry; HVIN: hyperventilation-induced nystagmus.

Reference [X]	Article Type Population	Clinical Data	Intervention	Results
Idriss et al. 2022 [11]	Case series (16 patients)	-HVIN+ (*n* = 6) -Sensorineural hearing loss (*n* = 4)	-Oxcarbazepine 300 mg/day -6 weeks (re-evaluation at 12 weeks)	-2 groups: nNIAC (*n* = 8), NVCC (*n* = 8). -8 children treated (4 in each group) -Substantial improvement (subjective). -Recurrence of vertigo when treatment was stopped <3 months (*n* = 4). -Auditory brainstem I–III interpeak latencies normalisation.
Lehnen et al. 2015 [12]	Case series; 16 patients, only 3 presented (8, 9, and 12 years old)	-Induced by movement or at rest (*n* = 2) -No trigger (*n* = 1) -Brief (seconds) -Several times a day -HVIN+ (*n* = 1) -Vestibular assessment: normal (*n* = 3) -PTA: normal (*n* = 3)	-Carbamazepine (50–100 mg/day) 2–4 mg/kg -In the evening	-Medication stopped at 6 months with no further attacks (*n* = 1) -Attempts to stop medication failed (4 and 18 months); asymptomatic at 3 years (*n* = 1) -Medication stopped after months, reappearance of symptoms, medication re-initiated efficiently (*n* = 1)
Hong et al. 2014 [13]	Case report (7-year-old girl)	-Rotatory vertigo once or twice an hour, 10 s. -Induced by running or moving. -HVIN+. -Caloric paresis (25%) -PTA normal	-Oxcarbazepine (300 mg/day) for one week -Then increased to 360 mg/day.	-Frequency of vertigo reduced by less than half. -Symptoms disappeared after one week, and the patient had been symptom-free for 5 months. The duration of treatment was not specified.
Nunez et al. 2024 [16]	Case report (16-year-old)	-Dizziness, unsteadiness; 20 s; 4 per week. -No trigger -EEG normal, MRI showed no NVCC	-Not alleviated by topiramate -Carbamazepine 200 mg/day (bid) -Unspecified duration	-Immediate and lasting response
Liu et al. 2023 [17]	Case report (22-year-old)	-Nonrotatory vertigo, a few seconds; 10 times a day -No trigger -HVIN− -PTA normal	-Carbamazepine 200 mg/day (bid) -2 months	-Symptoms completely abolished

## 4. Discussion

Given the paucity of research on sodium channel blockers for treating VP in children —with only case reports, a case series, and a retrospective study identified—a systematic review in the classical sense was not possible. Therefore, the present paper seeks to synthesize the arguments and justifications supporting treatment selection and the diverse clinical and electrophysiological findings in pediatric VP.

### 4.1. Sodium Channel Blockers for VP in Children

Carbamazepine (CBZ) and Oxcarbazepine (OXC) are the only sodium channel blockers reported for this indication in children. OXC was administered at 300–360 mg/day [11,13] and was well-tolerated. CBZ dosages ranged from 50 to 100 mg/day (or 2–4 mg/kg) in a single dose for younger children (age 8–12) [12] to 200 mg/day in two doses for older children [16,17]. While other sodium channel blockers have been used in adults (lamotrigine, lacosamide, etc.) [8], there are no reports of their use in children for this indication. Topiramate, tried for migraine in one instance, did not affect VP symptoms [16]. The duration of treatment was often not specified, hindering the establishment of guidelines, though a 6-week trial has been suggested [11]. Rapid effectiveness (within one week) was reported by Hong et al. (*n* = 1) and Lehnen et al. (*n* = 3) [12,13]. Lehnen et al. reported that treatment efficacy returned quickly upon restarting treatment after discontinuation [12]. In adults, arguments in favor of OXC over CBZ were presented [8]. OXC, a structural analogue of CBZ designed to reduce side effects, demonstrates in vitro sodium channel blockers at lower concentrations and exhibits less interaction with the cytochrome P-450 system. However, high-dose OXC can compromise oral contraceptive efficacy, and pregnancy safety data remain limited [18]. In France, CBZ is considered teratogenic. OXC is primarily excreted in the urine, requiring caution in severe renal impairment [19]. Unlike OXC, CBZ is mainly metabolized in the liver, which may necessitate dose adjustments. Furthermore, in France, OXC treatment does not routinely require biologic monitoring, unlike CBZ. Hence, OXC offers superior clinical advantages in terms of efficacy, tolerability, and drug interaction potential compared to CBZ in adults.

Microvascular decompression is a potential option for adults with VP who respond to but cannot tolerate sodium channel blockers, provided the affected side is clearly identified [6]. However, to the best of our knowledge, this surgical approach has not been reported in children with VP.

### 4.2. Diagnostic Elements

The diagnostic criteria for VP varied across the literature; articles published after 2016 adhered to the Bárány Society criteria [6], whereas earlier publications used the definition of Brandt et al. [1,4]. Potential triggers sometimes included physical exertion, though often no specific cause was identified [12,16,17]. The presence of spontaneous nystagmus was frequently reported; several mechanisms were proposed to explain the effects of the HVIN [11,12,13]. HVIN could be attributed to an improvement in nerve signal transmission in the partially demyelinated vestibular nerve, a phenomenon observed in other retrocochlear pathologies, such as vestibular schwannoma or vestibular neuritis. HVIN manoeuvre leads to modulation of spontaneous nystagmus, for instance, by reducing or reversing it [20]. Physiologically, the maneuver leads to a rise in the pH of the cerebrospinal fluid (due to a reduction in carbon dioxide levels). This change could reduce the amount of extracellular ionized calcium, which temporarily facilitates signal transmission in partially damaged nerve fibers [20]. On the other hand, the rapid change of nystagmus from an excitatory to a paretic pattern could also be related to the partial healing of demyelinated vestibular nerve fibers. Thus, another mechanism could take place, such as decompensation of the central mechanism initially put into play in the case of a progressive peripheral vestibular lesion.

Data on vestibular assessment in VP patients, particularly in children, are scarce and often derived from diverse, heterogeneous assessment methods. While adult vestibular evaluations often appear normal, some authors detected mild to moderate unilateral hypofunction during intercritical periods using tests such as cVEMP and ENG, among others [1]. Previous investigations showed saccular dysfunction (based on cervical VEMP findings) in 50% of the children in the VP group with NVCCSs [11]. Caloric testing showed a deficit in 34–50% of children in the combined study groups, and a reduction in video-head impulse test gain was noted in 25–37.5% of cases. Consistent with these findings, Hong et al.’s case report documented canal paresis through caloric testing [13].

Hearing loss is uncommon, typically mild, and usually affects one ear; associated auditory symptoms can sometimes indicate the affected side [6]. Unilateral staccato noises (typewriter nystagmus) have been reported in some adult patients with VP and may represent an additional diagnostic criterion for CVN “compression syndrome” (see [8]). To date, typewriter tinnitus has not been documented in the pediatric population, either in association with VP or independently. The importance of ABR for both diagnosing and monitoring VP was emphasized. Delayed auditory conduction and prolonged I–III interpeak latencies were found in 50% of children with nNIAC and 30% with NVCCSs [11]. Interestingly, OXC led to the normalization of these latencies in both groups. This suggests that ABR can be a valuable tool for diagnosis and follow-up (with or without treatment). However, it has been reported that long-term administration of antiepileptic drugs, especially CBZ, can delay auditory conduction in epilepsy (prolonging I–III interval) [21]. The normalization of this interval observed in VP patients treated with OXC may be due to the low doses and single-drug therapy compared to the higher doses and combinations often used for epilepsy.

Adapted MRI sequences with axial and coronal slices allow visualization of the CVN and rule out differential diagnoses. High-resolution IAC MRI with Constructive Interference in Steady State (CISS) or Fast imaging employing steady-state acquisition (FIESTA) brainstem sequences is suggested for diagnosing VP with NVCCSs [6,12], with axial and coronal slices being helpful [9]. Currently, a 7-Tesla MRI does not seem to offer a diagnostic advantage over a 1.5 or 3-Tesla MRI [7]. High-resolution computed tomography (HRCT) can evaluate the size of the IAC to identify bony variations affecting the CVN (see [9,10]) and to look for potential perilymph fistulas or other middle ear issues. Analysis of fusion images between high-resolution T2 MRI and HRCT of the temporal bones has been recommended in adults to better assess the CVN’s path and detect any deviations within the IAC in the axial or coronal planes [9,10].

### 4.3. VP Classification

According to a recent classification, the majority of VP cases are classical, involving vascular contact with the CVN [11,12,13,17]. The AICA was involved in all three cases of Lehnen et al.’s series [12] and two other case reports [13,17] as the likely compressing vessel. Secondary VP (nNIAC) was found in eight patients [11]. Nunez et al. presented the case of a young patient with typical VP symptoms but no NVCCSs on MRI [16]. This patient also had a WPW, a rare cardiac arrhythmia that is sometimes associated with Leber’s hereditary optic neuropathy, a mitochondrial genetic disorder that causes subacute blindness, mainly in young males [22]. The significance of this association in the context of VP and the typical Leber’s symptoms remains unclear; it is not known why this condition only affects the optic nerve, but the prevalence of brief dizziness would not necessarily be at the head of the symptoms. MRI normality could lead to the suspicion of idiopathic PV in this context; however, radiologic examination was not very detailed, and temporal bone tomodensitometry was not performed.

### 4.4. Differential Diagnoses for VP

The differential diagnoses of VP in children are those whose clinical descriptions correspond to brief, positional, stereotyped vertigo. The initial diagnostic workup in the presence of VP-like symptoms involves an MRI. The imaging modality is crucial for identifying potential NVCCSs as the underlying cause, as well as for excluding other possibilities such as schwannomas, and central diseases such as multiple sclerosis.

The most common cause of vertigo in children and adolescents is migraine, with more than half experiencing headaches [14]. BPVC is an episodic syndrome often linked to migraines and considered a possible precursor [23]. It is characterized by brief, recurrent attacks of vertigo, typically lasting only a few minutes and occurring without prodromes or warning signs [24]. BPVC usually begins before the age of 4 and resolves spontaneously by the age of 8 [25]. As BPVC episodes are usually infrequent and short-lived, and the condition resolves within a few years, treatment is generally unnecessary. The Barany Society recently proposed diagnostic criteria for “Vestibular Migraine of Childhood” (VMC) and probable VMC and introduced the term “Recurrent Vertigo of Childhood” (RVC), which is intended to replace the term BPVC. The criteria for VMC include (A) at least five episodes with vestibular symptoms of moderate or severe intensity lasting between 5 min and 72 h; (B) a current or past history of migraine with or without aura; and (C) at least half of the episodes are associated with at least one migraine feature. Probable VMC is diagnosed when at least three episodes are accompanied by at least one of criteria B or C from the VMC criteria. RVC is diagnosed when at least three episodes with vestibular symptoms lasting between one minute and 72 h are present, and none of the criteria B or C for VMC are met. In all cases, the condition must not be better explained by another headache disorder, vestibular disorder, or other condition [26]. Common migraine prevention medications for children include cyproheptadine, flunarizine, propranolol, and topiramate. While topiramate is frequently used for pediatric migraine, its effectiveness for VP has not been investigated, and gabapentin has shown limited success in VP [27]. Episodic ataxias are inherited channelopathies primarily affecting the cerebellum, displaying diverse genetic and symptomatic profiles. Their main feature is dizziness attacks lasting between 5 min and 12 h, often accompanied by walking difficulties, pallor, and slurred speech. Common triggers include physical activity, and gaze nystagmus and down-beat nystagmus can also occur [28]. The most prevalent type (type 2) is characterized by attack durations ranging from a few minutes to several hours, with over 90% of patients showing cerebellar signs [6].

Vertiginous epilepsy (VE) is also known as epileptic vertigo, vestibular epilepsy, or vestibular seizures. The seizures are thought to originate in the vestibular cortex, more specifically in parts of the parietal operculum and posterior insula [29]. According to Wood et al., any change in cognitive function or altered consciousness accompanying vertigo in children should prompt this diagnosis. Positional vertigo or hearing loss, on the other hand, are not diagnostic of VE, although auditory hallucinations may occur during temporal lobe activity. VE should be considered in patients with episodic vertigo and a family history of seizures [29]. In addition to MRI, evaluation includes electroencephalography (24 h EEG, if possible, given the short duration of seizures).

Benign paroxysmal positional vertigo (BPPV) is characterized by brief, intense episodes of vertigo triggered classically by abrupt changes in head position. Diagnosis is clinical, using adapted maneuvers. In children, BPPV is primarily described in cases of concussion [30] or as a concomitant secondary to any coexisting vestibular disorder [31,32]. According to Brodsky et al. [31], children with vestibular migraine or BPVC have a fivefold higher risk of BPPV recurrence compared to those without these disorders. In comparison with adults, the degree of nystagmus may be lower in pediatric patients, and recurrence may be lower [32]. Vertigo due to perilymphatic fistula or otic capsule dehiscence is usually triggered by the Valsalva maneuver or noises, and is accompanied by oscillopsia and instability. These attacks, which can last from a few seconds to a few days, can also occur with changes in head position and significant changes in altitude [6]. In Menière’s disease, positional nystagmus is possible, but attacks often last much longer.

## 5. Conclusions

Vestibular paroxysmia has been identified in pediatric patients and appears to respond to sodium channel blockers, such as oxcarbazepine and carbamazepine, in a manner similar to adults. To date, only a limited number of cases have been reported. There is a need to raise awareness of this treatable cause of episodic vertigo in children.

## Data Availability

No new data were created or analyzed in this study.

## Abbreviations

The following abbreviations are used in this manuscript:

AICA	Anterior inferior cerebellar artery
BPVC	Benign paroxysmal vertigo of childhood
CBZ	Carbamazepine
CVN	Cochleo-vestibular nerve
HVIN	Hyperventilation induced nystagmus
IAC	Internal auditory canal
MRI	Magnetic resonance imaging
nNIAC	Near narrowed internal auditory canal
NVCC	Neurovascular cross compression
OXC	Oxcarbazepine
PTA	Pure tone audiometry
VE	Vestibular epilepsy
VP	Vestibular Paroxysmia
WPW	Wolff Parkinson White
